# Autoimmune Complications after Hematopoietic Stem Cell Transplantation in Children with Nonmalignant Disorders

**DOI:** 10.1155/2014/581657

**Published:** 2014-01-19

**Authors:** Abdalla Khalil, Irena Zaidman, Reuven Bergman, Ronit Elhasid, Myriam Weyl Ben-Arush

**Affiliations:** ^1^Department of Pediatric Hematology Oncology, Meyer Children's Hospital, Rambam Health Care Campus, P.O. Box 9602, 31096 Haifa, Israel; ^2^Department of Dermatology, Rambam Health Care Campus, Haifa, Israel; ^3^Faculty of Medicine, Technion-Israel Institute of Technology, Haifa, Israel; ^4^Pediatric Hematology Oncology Department, Dana Children's Hospital, Tel Aviv, Israel

## Abstract

*Background*. Hematopoietic stem cell transplantation (HSCT) remains the only curative treatment for many nonmalignant disorders, such as autoimmune disorders, inborn metabolic disorders, hemoglobinopathies, and immunodeficiency disorders. Autoimmune complications (AICs) after HSCT, such as autoimmune cytopenias, autoimmune hepatitis, primary biliary cirrhosis, and autoimmune cutaneous manifestations, are still neither well defined nor characterized. *Patients*. Between 2000 and 2012, 92 patients (47 males, 45 females) were treated with HSCT in our hospital, 51 with congenital hemoglobinopathies, 19 with primary immunodeficiency disease, 10 with metabolic disorders, five with Fanconi anemia, three with aplastic anemia, and four with familial hemophagocytic lymphohistiocytosis. *Results*. Mean age at HSCT was 6.4 years (range, 0.2–32 years) and mean duration of followup after HSCT was 6.81 years (range, 1–11 years). Sixteen (17.4%) patients developed chronic GVHD and five (5.4%) showed sclerodermatous features. Five (5.4%) patients were diagnosed with scleroderma manifestations, six (6.5%) with vitiligo, six (6.5%) with autoimmune hemolytic anemia (AIHA), six (6.5%) with idiopathic thrombocytopenia, three (3.3%) with mild leucopenia, two (2.2%) with aplastic anemia, two (2.2%) (one boy, one girl) with autoimmune thyroid disease, and one (1.1%) with autoimmune hepatitis. *Conclusions*. It was concluded that AICs are clinically significant complications after HSCT that contribute to morbidity but not to mortality. AICs are more frequent after HSCT for metabolic disorders, and sclerodermatous GVHD is more significant in children who underwent allogeneic HSCT for hemoglobinopathies. The potential to identify risk factors for AICs could lead to less morbidity and mortality and to maintain the patient's quality of life.

## 1. Introduction

Hematopoietic stem cell transplantation (HSCT) is a therapeutic option for a number of non-malignant disorders, such as autoimmune disorders [1], inborn metabolic disorders [2], hemoglobinopathies [3], and immunodeficiency disorders (IDD) [4]. Autoimmune complications (AICs) after HSCT are still not well characterized but have multifactorial occurrence, such as autoimmune cytopenias (AICPs), autoimmune hepatitis (AIH), and autoimmune thyroid disease (AITD).

Chronic graft-versus-host disease (GVHD) mimics some autoimmune diseases such as scleroderma. Other AICs have been reported in case reports or short series and their pathophysiology remains poorly understood.

Most AICs seem to be related to a poor or inadequate immunologic recovery after HSCT with an imbalance between autoregulatory and autoreactive lymphocytes.

The aim of this study was to analyze the incidence and risk factors for the development of AICs, as well as their prognosis and response to treatment in patients undergoing allogeneic HSCT for non-malignant disorders who were followed up and treated at Rambam Health Care Campus in Haifa, Israel. Furthermore, this report assesses the prevalence of AICs, survival, and prognosis after HSCT for non-malignant disorders.

## 2. Patients and Methods

We reviewed the clinical charts of all the patients who underwent allogeneic HSCT for non-malignant disorders between 2000 and 2012, using various sources of stem cells harvested from matched family donor, matched unrelated donor, and umbilical cord blood (CB). Ninety-two patients were analyzed (47 males, 45 females), of whom 51 were diagnosed with congenital hemoglobinopathies (HGP), 19 with primary immunodeficiency disease (PID), 10 with metabolic disorders, five with Fanconi anemia (FA), three with aplastic anemia (AA), and four with familial hemophagocytic lymphohistiocytosis. Mean age at HSCT was 6.4 years (range, 0.2–32 years), and mean duration of followup after HSCT was 6.81 years (range, 1–11 years).

Twenty-four thalassemic patients were classified as class III, 21 as class II, and six as class I. Eleven patients were splenectomized and mean ferritin level was 2859 ng/mL (350–10,900). Seven patients underwent a second HSCT after a first transplant rejection (*n* = 6) or aplastic anemia (*n* = 1).

Ninety patients had an HLA-matched donor, all selected using high-resolution molecular typing of both HLA class I and II loci. All donor-recipient pairs had complete identity for all HLA class I (i.e., A, B, and C) and class II loci (i.e., DRB1, DRB3, DRB4, DRB5, DQA1, and DQB1). Eighty-five had related donors and seven had unrelated donors, while 55 received peripheral blood stem cells (PBSCs). One patient received haploidentical PBSC, 25 received bone marrow (BM), and 11 CB stem cells (one was mismatched by one antigen). Sixty-seven (73%) patients had full donor chimerism and 25 (27%) had stable mixed donor chimerism (MC) (range, 17–98%). All cases of MC had related HSCT. The diagnosis of AIC was made by standard laboratory-clinical evidence and denial of other diseases.

## 3. Results

**Figure 1:**
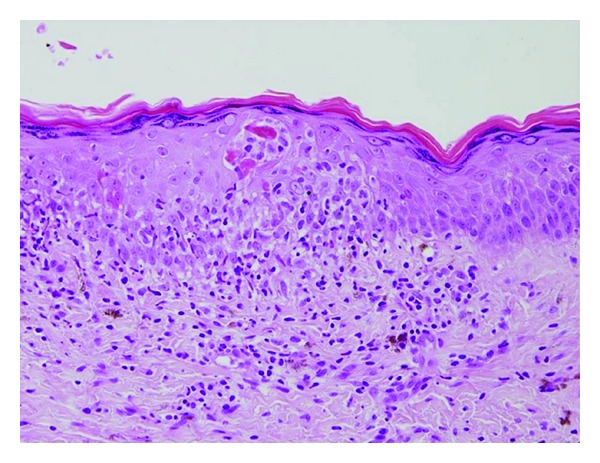


**Figure 2:**
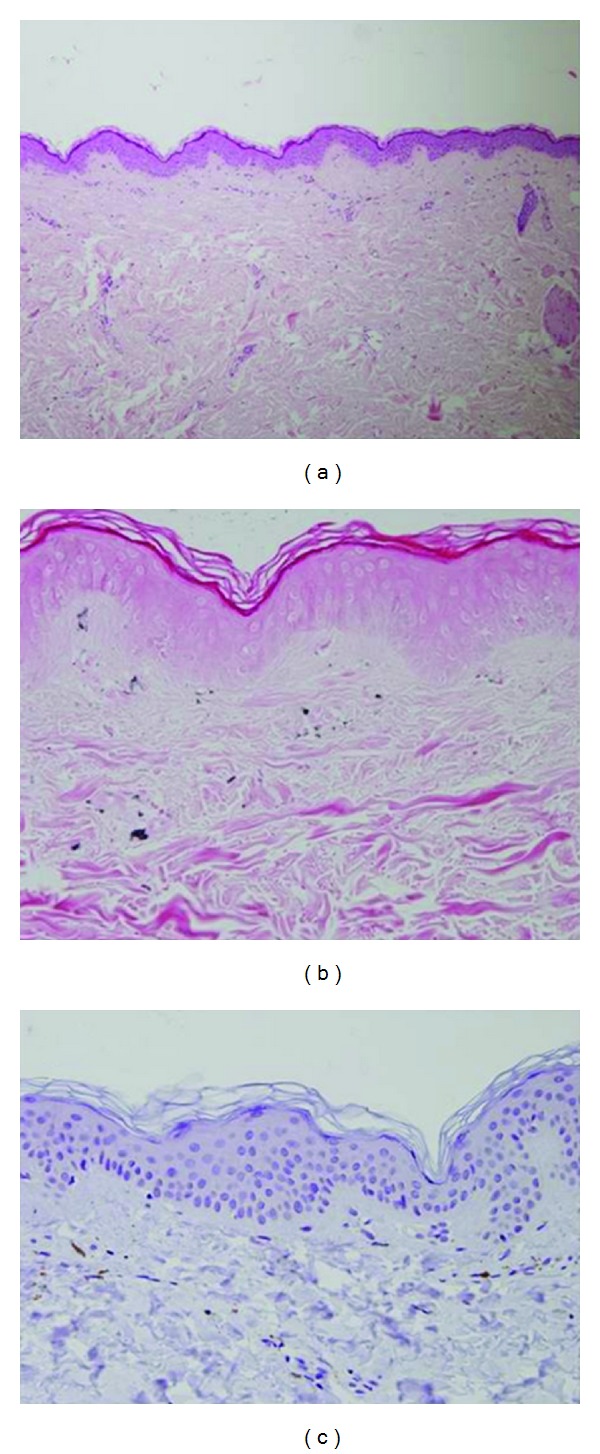
(a) Normal looking epidermis and dermis except that there is a lack of melanin pigment in the epidermis. (H&E ×100). (b) Fontana stain shows lack of melanin pigment in the epidermis and several melanophages in the papillary dermis (×400). (c) MART-1 immunostain shows lack of melanocytes in the epidermis (×400).

Table 1 summarizes AICs after HSCT for non-malignant disorders. At a mean age of 15.3 years (range, 6–24.5 years), at a median of 10 months after HSCT (range, 3–22 months), seven (7.85%) patients were diagnosed with autoimmune cutaneous manifestations—two developed areas of vitiligo only, five had skin sclerodermatous lesions (four localized and one generalized)—and all had peripheral stem cell sources (one unrelated, the others related PBSC). Five patients were transplanted for HGP and two for congenital PIDs. Lung involvement occurred only in one patient. Of the 92 patients analyzed, 16 (17.4%) developed chronic GVHD and five (5.4%) showed sclerodermatous features. However, none had autoimmune markers such as anti-nuclear antibodies (ANA), antismooth muscle, and scleroderma (Scl-70) antibody. Histological examination was necessary to make the diagnosis in three patients, which showed histopathological features of scleroderma. One patient had an acute GVHD rash with histological findings of lymphocytes infiltrating the epidermis, leading to numerous apoptotic epidermal cells (Figure 1). Two years after the resolution of this rash, vitiligo appeared in the skin with histological evidence of total disappearance of the melanocytes (Figure 2). Sclerodermatous lesions were defined as generalized if more than two anatomic sites were involved.

*AICP*. AIHA was diagnosed with positive direct Coombs' test with clinical and laboratory significant hemolysis and the exclusion of other causes of immune hemolytic anemia. Six (6.5%) patients were diagnosed with AIHA at 10 months after HSCT. Four of the six patients transplanted for HGP developed mild-to-moderate anemia, responsive to standard therapy. The other two patients transplanted for metabolic disorders developed severe AIHA, one unresponsive to standard and salvage therapy.

Six (6.5%) patients were diagnosed with idiopathic thrombocytopenia. Five of the six patients were transplanted for HGP and one for PID. Two patients needed IV immunoglobulins (IVIG) and corticosteroid therapy, and two patients had mild, stable chronic thrombocytopenia. Three patients developed mild stable leucopenia.

Two patients were diagnosed with graft failure 1.1 years after HSCT (for thalassemia major and FA). One patient needed a second HSCT and the other patient responded well to corticosteroid therapy.

*AITD*. Two patients (one boy, one girl) were diagnosed with autoimmune hypothyroidism (elevated TSH and anti-thyroglobulin antibodies, low FT4 levels). One AITD was associated with AIHA one year after HSCT; the other case was associated with mild thrombocytopenia 10 years after HSCT. No patient developed GVHD.

One patient was diagnosed with AIH one year after HSCT. AIH was diagnosed on histological examination and the exclusion of other causes.

## 4. Discussion

This study shows that AIC is a clinically significant and common complication after HSCT for non-malignant disorders. HSCT can result in AID to the recipient or it may concomitantly cure another autoimmune disease in the recipient. If the basic defect of an autoimmune disease is within the stem cells, it may be cured by allogeneic HSCT while, if the primary defect is an aberrant immune reaction to an acquired or a self Ag, tolerance may be acquired in the newly reconstituted immune system after HSCT [1].

Practical problems were encountered by clinicians caring for patients with chronic GVHD and AICs. Clinical manifestations of chronic GVHD are similar to autoimmune collagen vascular diseases [5], such as scleroderma, morphea, polyserositis [6], obstructive liver disease,a and obstructive pulmonary disease.

Cytopenias are the most common AIC after HSCT, such as autoimmune neutropenia (AIN), immune thrombocytopenia (IT), and autoimmune hemolytic anemia (AIHA), or a combination of two of these disorders with pancytopenia. Cytopenia following HSCT may occur via alloimmune mechanisms when host or donor immunity reacts against donor or host elements, respectively, or autoimmune when donor immunity reacts against donor hematopoietic tissue [7]. Alloimmune cytopenias have been reported primarily after allogeneic HSCT, whereas AICPs have been reported following both allogeneic and autologous HSCT. AICP remains as a diagnosis of exclusion of other causes (drugs, infections, and GVHD), with a normal appearance of the affected lineage in the bone marrow, and the presence of auto- or allo-antibodies in the serum. Alloimmune cytopenia may be related to major or minor mismatches in the ABO or RH system.

All our cases of cytopenia were nonsplenectomized, received related PBSC, and had no GVHD association. One case of ITP received a major ABO-mismatched graft for aplastic anemia, and two cases of ITP received a sex-mismatched graft. ITP has been reported early after allogeneic HSCT [8]. It is not clear whether cytopenia is related to the process of HSCT or to autoimmune dysregulation. Autoimmune mechanism of cytopenia after HSCT is probably the main cause of IT [9]. IT can also be caused occasionally by the presence of a donor origin autoantibody [10] or by the presence of a recipient-origin alloantibody against a donor platelet alloantigen.

In our series, AIHA was a relatively uncommon complication after HSCT for non-malignant disorders. The incidence of pediatric AIHA after HSCT is not unknown, as there have been isolated case reports [11]. Chen et al. [12] reported an incidence of 3.1% of autoimmune hemolysis among 293 patients after HSCT.

Our patients underwent HSCT for metabolic disorders (X-linked adrenoleukodystrophy, MNGIE disease), and one received matched unrelated umbilical stem cells, mismatched in the RH system. Despite the normal numbers of B lymphocytes, he continues to have hypogammaglobulinemia with low IgG levels and requires monthly IVIG infusions. The other patient received PBSC with HLA, ABO, and sex-matched. There was no GVHD. Time from HSCT to AIHA was 7.5 months, similar to that reported [13]. AIHA can be subclassified according to antibody temperature reactivity, such as warm (IgG), cold (IgM), or biphasic hemolysis. Significant AIHA often results from warm IgG autoantibodies. One of our patients presented warm IgG autoantibodies, unresponsive to standard and salvage therapy. The other patient presented cold agglutinins (IgM autoantibodies) with good response to immunosuppressive and corticosteroid treatment. AIHA may be related to an unrelated donor or to major or minor mismatches in the ABO or RH system. O'Brien et al. [13] showed that metabolic disorders were the only factor significantly associated with the development of AIHA following unrelated donor transplant. However, the occurrence of AIHA was previously reported following unrelated UC blood transplantation [13]. Unlike the report by Horn et al. [14], we did not find an increased incidence of AIHA after HSCT among patients who received T-cell depleted grafts or in patients with PID undergoing mismatched HSCT with complete T-cell depletion. The possibility of AICP should be considered when peripheral cytopenia or hemolysis develops after HSCT. The diagnosis is supported by a normal appearance of the affected lineage or lineages in the bone marrow, the absence of other apparent causes for the cytopenia, and the presence of the relevant antibodies in the serum. However, any of these features may be absent in individual cases.

Au et al. [15] reported the occurrence of AITD after HSCT. In our series, the first patient developed AITD associated with AIHA one year after HSCT for MNGIE disease. The second patient underwent HSCT for WAD and developed AITD 10 years after HSCT. It was associated with mild thrombocytopenia and hypogammaglobulinemia with low IgG levels which required long-term (7.5 years) monthly IVIG infusions. Both patients had not received any radiation and did not have GVHD. In AITD, in addition to thyroid antibodies, patients can have antibodies to other tissues or enzymes as well, putting them at a higher risk to develop another autoimmune condition. In our series, the association between AITD and AIHA suggests the impairment of regulatory mechanisms of the immune system. AITD may develop on its own or may be associated with other autoimmune disorders, such as AIHA, hypogammaglobulinemia, or IT. AITD after HSCT can occur by the mechanism of direct transfer of autoreactive cells from donor to recipient (adoptive autoimmunity) [16]. The pathogenesis of AITD is complex. However, the main mechanism responsible for hypothyroidism secondary to AITD is believed to be the direct killing of thyroid cells by cytotoxic T cells [17].

Karthaus et al. [18] reported that even low numbers of lymphocytes are capable of transferring autoimmune disorders post-HSCT, while most of the cases can be attributed to the immunologic imbalances characterizing the after HSCT period. Factors that may expose to AICs after HSCT include genetic predisposition, environmental factors such as infections, and autoimmune manifestations related to the donor.

AIH after HSCT is rare, especially in children, and should be considered a long-term complication that follows a chronic and progressive but sometimes acute and fulminant form [19]. AIH in children has been reported in case reports after liver or HSCT [20, 21]. The etiology remains obscure but may be related to an autoimmune process, triggered by an environmental or intrinsic factor [22]. However, Sherer et al. noted that autoimmune disease can be induced bythe transplantation procedure [23]. It may be developed by an immune system dysregulation due to an adequate function of suppressor T cells in the post-HSCT period, delayed recovery of regulatory T cells, or activation of autoreactive T cells. We did not find an association of AIH with cyclosporine as reported by Damoiseaux and Van Breda Vriesman [24] or with hereditary metabolic disorders, such as hemochromatosis and Wilson's disease. We also failed to note an association with the HLA haplotypes: B8, B14, DR3, DR4, and Dw3 [22]. Habib et al. [25] speculated that a multistep sequence of events may also underlie the state of antiliver reactivity that resembles AIH after HSCT. AIH is a heterogeneous disorder that can be divided into two types, depending on the presence of autoantibodies [26]: Type 1 is associated with the presence of ASMA or ANA for about 75% of patients [16]; Type 2 is associated with the presence of either anti-LKM-1 or anti-liver cytosolic-1 antibodies [19]. One of our patients presented AIH without GVHD association or circulating autoantibodies as “autoantibody-negative AIH.” Liver biopsy established the diagnosis of AIH: portal eosinophilic and plasma cell infiltration, (no lymphocytic and granulomatous infiltrates of bile duct; ductopenia or other pathological changes), and the patient responded well to corticosteroid therapy.

Cholestasis predominates the clinical presentation of chronic liver GVHD. However, isolated liver involvement by chronic GVHD, without the clinical symptoms of other organ involvements, or histopathologic findings on biopsy is uncommon.

Autoimmune cutaneous manifestations are uncommon in pediatric patients, although there have been several reports occurring after HSCT complicated by GVHD. Cho et al. [27] hypothesized that GVHD may trigger an autoimmune cascade leading to the development of vitiligo. In our series, four patients were diagnosed with vitiligo after GVHD onset, all of whom received PBSC for HGP. The other two cases were transplanted for PIDs (XLP, Omenn syndrome); one received haploidentical PBSC with complete donor chimerism, while the other received unrelated CB and had MC. There was no GVHD and no autoimmune features before HSCT. Vitiligo may be a *de novo* development in a genetically predisposed patient or an autoimmune phenomenon in association with GVHD [23]. Ogg et al. [28] noted melanocyte-specific cytotoxic T lymphocytes with a high frequency in patients with vitiligo. Its association with other autoimmune diseases and the demonstration of melanocyte-specific antibodies [29] in some patients support an autoimmune etiology. The association found between vitiligo *de novo* and PIDs is not surprising. PIDs can impair negative regulation of immune responses that may predispose to autoimmunity. It is difficult to demonstrate which part of the immune system affects the melanocytes, because its pathogenesis is obscured. Prior reports suggested two possible explanations for the presence of melanocyte-specific T cells: direct transfer from donor or *de novo* development.

Sclerodermatousr lesions after HSCT have been described in small series and recently considered as a form of sclerodermatous GVHD [30]. In our study, the rate of sclerodermatous manifestations was higher (7.8%) than that published (3.6%) [31]. We found that sclerodermatous GVHD was more likely to develop in children who underwent HSCT for HGP, perhaps owing to the relatively different patient ages at HSCT and to the fact that all of our four patients received PBSC. We found no difference between conditioning with or without antithymocyte globulin. The pretransplant serologic status of cytomegalovirus (CMV) in the recipient is associated with an increased probability of chronic GVHD [32] and could potentially increase the development of AICs. In our study, most patients (5/7) developed autoimmune cutaneous manifestations associated with CMV reactivation, whereas this association could not be confirmed in other AICs.

Autoantibodies are an expression of B-cell hyperactivity promoted by autoreactive T cells in autoimmune disease and by donor T cells in chronic GVHD [28]. In our group, the generalized scleroderma form presented a poorer outcome than the localized form probably because of multiorgan involvement. All patients with localized scleroderma responded partially to immunosuppressive therapy except one. Sclerodermatous lesions after HSCT have a broad clinical spectrum and present therapeutic challenges [33].

None of the children in our cohort who underwent HSCT for correction of metabolic disorders developed autoimmune cutaneous manifestations.

Children with transfusion-dependent anemias, such as thalassemia and sickle-cell disease, frequently develop iron overload due to RBC transfusions, ineffective erythropoiesis, and intestinal absorption of iron. The risk of cardiac disease and early death is increased in patients with serum ferritin of more than 2500 ng/mL [34]. Children with thalassemia who have not received adequate iron chelation are also prone to developing and dying from infections. The relationship between iron overload and GVHD and AICs also needs to be further elucidated. Iron might increase the risk of acute and chronic GVHD through its tendency to cause direct organ toxicity; alternatively, it might decrease the risk of GVHD through its ability to impair host immune responses.

Our findings are limited by the relatively small number of patients, and the retrospective study cohort is a mix of pediatric and adult patients because these diseases are illnesses that are diagnosed in children who underwent bone marrow transplantation when they were under the age of 18 years and continued followup in our department.

## 5. Conclusions

In summary, our study suggests that patients tend to develop AICs after HSCT for non-malignant disorders. AICs are a clinically significant complication after HSCT that contribute to morbidity but not to mortality. Cytopenias are the most common AIC. AIHA, AITD, and AIH are more significant after HSCT for metabolic disorders, and autoimmune cutaneous manifestations are more frequent in children who underwent allogeneic HSCT for HGP. The potential to identify risk factors for AICs could lead to less morbidity and mortality and to maintaining quality of life.

## Figures and Tables

**Table 1:** Patient characteristics.

Indication for HSCT	Metabolic disorders	PIDs	HGP	FHL	Fanconi and SAA	Risk factors
Number of Patients	10	19	51	4	8	
AIC, *n* (%)						
AICP ITP + thrombocytopenia		1 (5.3)	5 (9.8)			ABO and sex-mismatched graft, metabolic disorder
Leukopenia		1 (5.3)	2 (3.9)			
AIHA	2 (20)		4 (7.8)			
AA			1 (1.96)		1 (12.5)	
AITD	1 (10)	1 (5.3)				Hypogammaglobulinemia metabolic disease, association with other ILCs
AIH	1 (10)	0	0	0	0	Metabolic disease
Scleroderma	0	0	5 (9.8)	0	0	HGP, age, chronic GVHD, PBSC, unrelated donor
Vitiligo	0	2 (10.5)	4 (7.8)	0	0	

PID: primary immunodeficiency disorder; HGP: hemoglobinopathy; FHL: familial hemophagocytic lymphohistiocytosis; AIC: autoimmune complication; AICP: autoimmune cytopenia; ITP: idiopathic thrombocytopenic purpura; AIHA: autoimmune hemolytic anemia; AA: aplastic anemia; AITD: autoimmune thyroid disease; AIH: autoimmune hepatitis.
